# Penetrating Aortic Ulceration Treated with Castor Branched Aortic Stent Graft—A Case Series

**DOI:** 10.3390/ijerph19084809

**Published:** 2022-04-15

**Authors:** Antonio Rizza, Francesco Negro, Stefano Palermi, Cataldo Palmieri, Michele Murzi, Giovanni Credi, Sergio Berti

**Affiliations:** 1 Cardiology Unit, Fondazione Toscana Gabriele Monasterio, 54100 Massa, Italy; rizza@ftgm.it (A.R.); palmieri@ftgm.it (C.P.); berti@ftgm.it (S.B.); 2 Human Anatomy and Sport Medicine Division, Department of Public Health, University of Naples Federico II, 80131 Naples, Italy; stefanopalermi8@gmail.com; 3 Cardiac Surgery Department, G. Pasquinucci Heart Hospital, 54100 Massa, Italy; michelem@ftgm.it; 4 Unit of Vascular Surgery, Hospital of Massa Carrara, 54100 Massa, Italy; giovanni.credi@uslnordovest.toscana.it

**Keywords:** acute aortic syndromes, penetrating aortic ulceration, aortic arch, left subclavian artery, endovascular graft

## Abstract

Penetrating aortic ulceration (PAU) is an acute aortic syndrome similar to aortic dissection and intramural hematoma. It is the result of a tunica intima ulceration due to atherosclerotic disease. This clinical condition can lead to serious complications and a poor short-term prognosis, especially in high-surgical-risk patients. We report three cases of patients referred to “Ospedale del Cuore” of Massa (Italy) with PAU at the aortic arch-proximal descending aorta level who could not undergo surgical intervention. For the first time in Italy, we successfully treated these patients with a full percutaneous implantation of a Castor branched aortic stent graft. Our case series shows that this type of endovascular graft is an effective, safe, and feasible treatment for PAU involving a distal aortic arch and avoiding surgery and related complications.

## 1. Introduction

A penetrating aortic ulceration (PAU) consists of an ulceration in an atheromatous plaque that extends deeply through the intima and into the aortic media. It may precipitate an intramedial dissection (usually localized) or may rupture into the adventitia to form a pseudoaneurysm [1]. It can be diagnosed with a computed tomographic angiography (CTA) with a limited-contrast effusion overcoming the aortic tunica intima [2]. There are often multiple aortic ulcers that may vary greatly in size (ranging from 5 mm in diameter and 4–30 mm in depth). PAU is an acute aortic syndromes (AAS) consisting of aortic dissection (AD), intramural haematoma (IMH) and aortic truma. PAU’s classification shows important similarities with the Stanford aortic dissection classification. When PAU is located in the ascending aorta, we refer to Type A PAU. Conversely, when PAU is located in the descending thoracic aorta or abdominal aorta, we refer to Type B PAU. In 1999, Svensson et al. [3] proposed a new AAS classification: class I is represented by classical AD, class II by intramural hematoma or haemorrhage, class III by subtle dissection without hematoma, class IV by penetrating aortic ulcer, and class V by iatrogenic or traumatic aortic dissection.

Typically, PAU affects older patients (>70 years) and those with extensive and diffuse atherosclerotic disease than those with AD. The natural history of PAU includes the formation of medial haematoma, dissection, and/or adventitial false aneurysm, and transmural rupture. Compared with aortic dissection, the risk of rupture (7% for Type A and 4% for Type B AAD) is considerably higher (up to 40% for PAU) [4].

In some cases, PAU may be clinically silent, as some findings identified through imaging; otherwise, the most frequent clinical presentation is pain that can be reported in the chest, back or abdomen. This clinical presentation overlaps with classical AAD. PAU’s natural history can be benign, without disruption progression, or it can evolve into a saccular pseudoaneurysm. Symptomatic, extensive, and aneurysmal PAU is thought to have an increased risk of rupture, and thus the effective management of PAU is needed.

Among imaging modalities, contrast-enhanced CT, including axial and multiplanar reformations, is considered the diagnostic technique of choice [5].

Frequently adopted interventions include radiological surveillance, open surgical repair and thoracic endovascular aortic repair (TEVAR). Although asymptomatic patients may be conservatively managed, some ulcers become aneurysmal or grow in size [6]; it is estimated that 30% of medically managed PAU worsens [7]. Some case series showed that 14% of those that were conservatively managed at first subsequently required surgical repair for aneurysmal degeneration [8].

Regardless of interventional management, the initial medical therapy of all AAS should aim to decrease wall stress in order to limit the extension of the dissection and reduce the risk of developing end-organ damage and rupture. In particular, it is important to obtain an adequate control of pain (intravenous opiate analgesia), heart rate (<60 bpm) and blood pressure (systolic blood pressure between 100 and 120 mmHg). Betablockers (propranolol, metoprolol, labetalol, or esmolol) represent first-line drugs. Non-dihydropyridine calcium channel antagonists (verapamil and diltiazem) are reasonable alternatives for patients truly intolerant to beta-blockers. In case of poor blood pressure control, intravenous vasodilators such as sodium nitroprusside may be used [5].

At present, it is recommended to treat Type A PAU with surgery, while medical therapy with careful clinical follow-up and imaging surveillance represents the first choice for Type B PAU management. On the other hand, an interventional approach is recommended in complicated Type B PAU (signs of aortic rupture); endovascular stent grafting is usually preferred to surgical repair considering lesions’ segmental nature (anatomical landmarks) and patients’ increased risk profiles [5].

When PAU (or B type AD) is located near the left subclavian artery (LSA), the most pursued strategy consists of performing a carotid-subclavian bypass followed by TEVAR, not accounting for the emergence of LSA. In this case, the patient has to undergo two different procedures (the first of which is a surgical intervention). When this occurs in high-surgical-risk patients, the prognosis worsens, but some Asian patients were subject to the use of a branched aortic stent graft that could avoid surgical procedures. In Italy, there was no experience of this kind of endovascular graft. Therefore, the aim of this case series is to show this intervention and its advantages in detail.

## 2. Case 1

**Figure 1:**
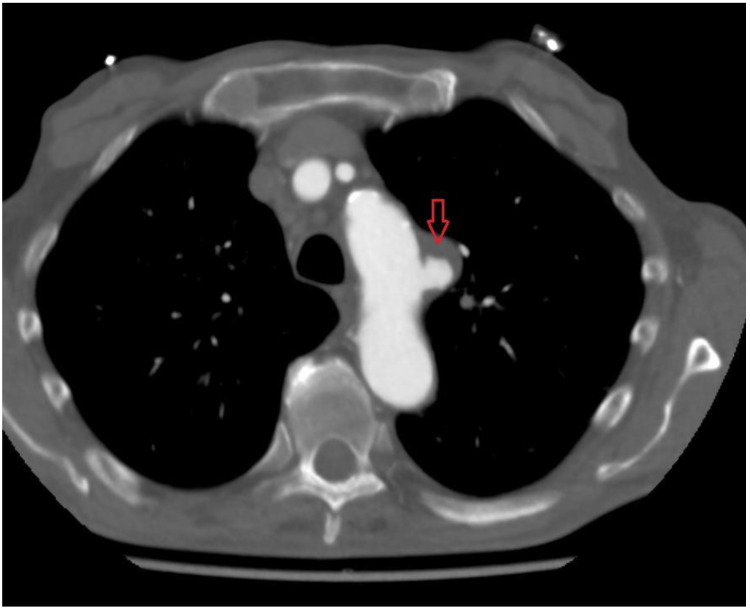
Preprocedural CT scan; red arrow shows PAU.

**Figure 2:**
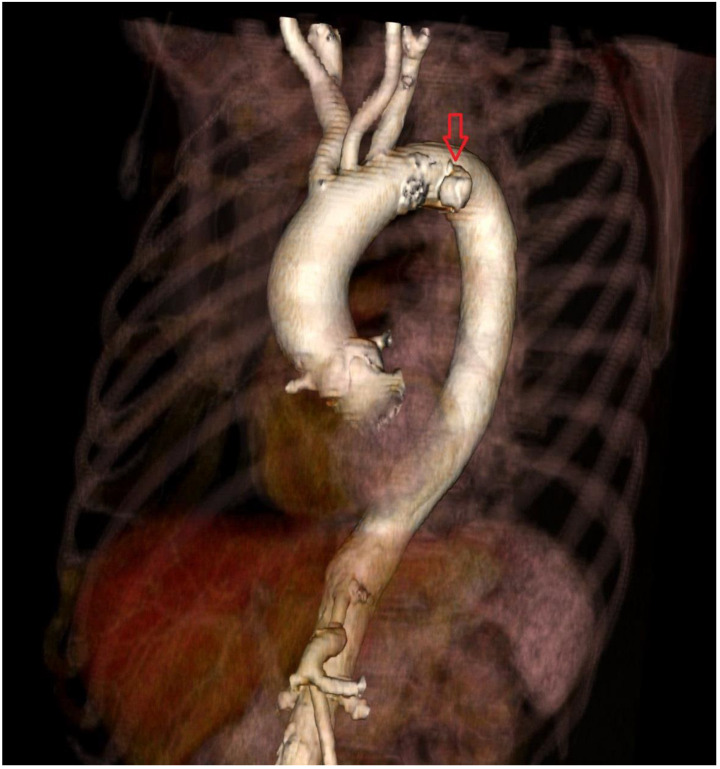
Preprocedural 3D CT; red arrow shows PAU.

A 79-year-old man with arterial hypertension, carotid artery stenosis and previous subdural hematoma presented in our hospital with an episode of transient chest pain radiating to his back; computer tomography showed this to be a penetrating aortic ulceration. After 2 years of follow up, the last computed tomography (CT) revealed intimal ulceration in the aortic arch. The lesion, a penetrating atherosclerotic ulcer, affected the left aspect of the patient’s aortic arch with diameter of 46 mm × 22 mm and height of 21 mm (Figure 1 and Figure 2), then the risk of progression to frank aortic dissection was large. Both the innominate artery and the left carotid artery were patent and free of dissection. The patient’s surgical risk was calculated as moderate–high, and thus we decided to perform an endovascular approach.

The procedure was performed under general anesthesia; a 6 F pigtail was advanced through a right radial arterial access into an ascending aorta for aortic angiographies; then a 10 F right femoral arterial access was obtained with the pre-implantation of two percutaneous closure systems, subsequently upgraded to 18 F; afterwards, an 8 F left brachial arterial access was obtained; a guidewire was advanced through the brachial access into the femoral access, forming a brachial–femoral loop; a dedicated catheter was advanced on the guidewire; then, an extrastiff guidewire (0.035″) was advanced through femoral access to the ascending aorta; the branch guidewire was positioned in the catheter introduced from the brachial access; and a Castor 34 mm × 30 mm × 200 mm stent graft with a 12 mm × 25 mm branch for LSA was positioned and released. Immediate aortography confirmed the patency of the side branch, the absence of endoleaks and PAU exclusion.

The therapy at discharge included amlodipine 5 mg, ramipril 2.5 mg, a combination of low-dose aspirin and clopidogrel (for 3 months, followed by low-dose aspirin only).

**Figure 3:**
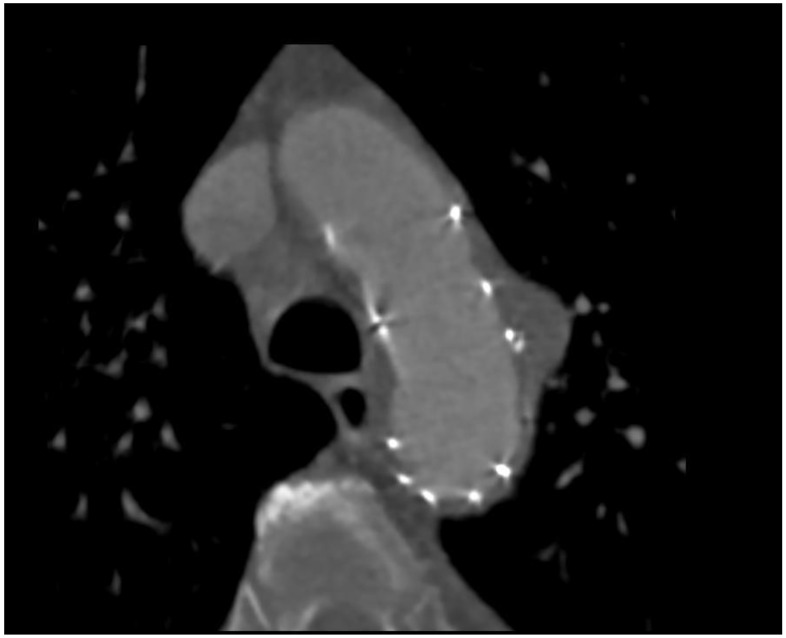
Postprocedural CT scan.

**Figure 4:**
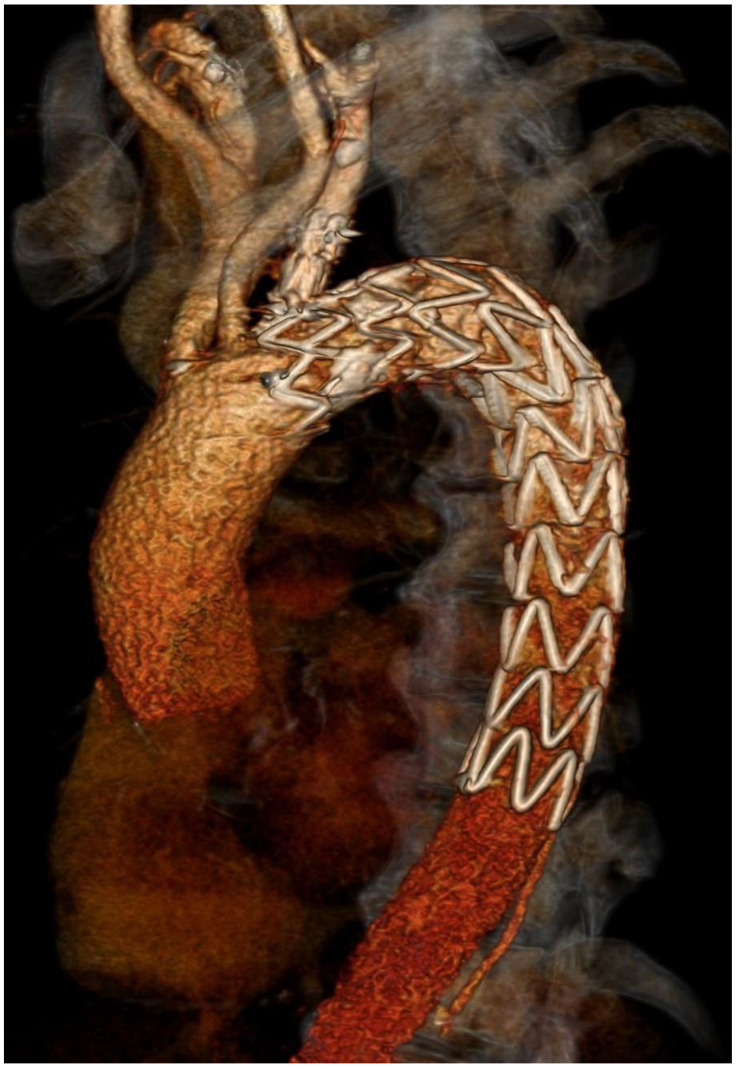
Postprocedural 3D CT showing Castor stent graft.

Finally, CT angiography performed after 24 h showed good results for endovascular prosthesis implantation (Figure 3 and Figure 4).

After a follow-up of 1 year, the patient was in good functional status and CT angiography showed proper endoprosthesis expansion without thrombotic apposition.

## 3. Case 2

A 61-year-old male patient with a history of smoking and dyslipidemia had PAU that was incidentally discovered during an angio-CT performed for a stroke involving the right middle cerebral artery and treated with fibrinolysis. After a thromboendoarterectomy of the right common carotid artery, the patient repeated an angio-TC that confirmed the presence of PAU at the proximal descending aorta, 5 mm distal to the origin of LSA. The surgical risk was low but, considering of the absence of aortic disease-related symptoms, the case was discussed by the heart team, and we decided to perform a minimally invasive implantation of a branched stent graft.

The procedure was performed under general anesthesia. A 6 F pigtail was advanced through a right radial arterial access into an ascending aorta for aortic angiographies. Then a 10 F right femoral arterial access was obtained with the pre-implantation of two percutaneous closure systems and subsequently upgraded to 18 F. Afterwards, an 8 F left brachial arterial access was obtained; a guidewire was advanced through the brachial access into the femoral access, forming a brachial-femoral loop, and a dedicated catheter was advanced on the guidewire. Then, an extrastiff guidewire (0.035″) was advanced through femoral access to the ascending aorta, and the branch guidewire was positioned in the catheter introduced from the brachial access; a Castor 34 mm × 34 mm × 150 mm stent graft with a 12 mm × 25 mm branch for LSA was positioned and released at the PAU level. A final angiography showed the exclusion of PAU and absence of endoleaks. There were no major complications at the vascular accesses level.

The therapy at discharge was ramipril 2.5 mg, low-dose aspirin, plus clopidogrel (for 3 months, followed by low-dose aspirin only).

The patient performed a CT angiography after 1 year that showed good results with no evidence of endoleaks, strut malapposition or thrombosis.

## 4. Case 3

A 62-year-old male patient with a history of smoking and arterial hypertension had an incidental finding of a focal saccular 27 mm × 26 mm × 15 mm aneurysm during an angio-CT performed in the emergency department for a thoracic trauma. The patient was referred to our hospital and an angio-CT confirmed the presence of this PAU with a parietal thrombotic apposition, localized in the ventral portion of aortic arch. Although the surgical risk was not impossible, the patient had initial findings of ascending aorta dilatation and had no symptoms; therefore, we decided to avoid a surgical intervention and opted for a full percutaneous approach.

**Figure 5:**
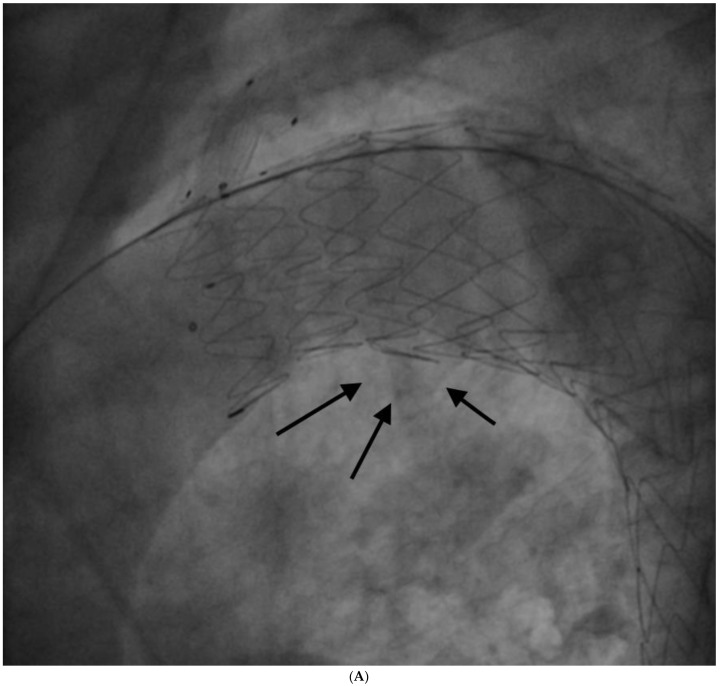

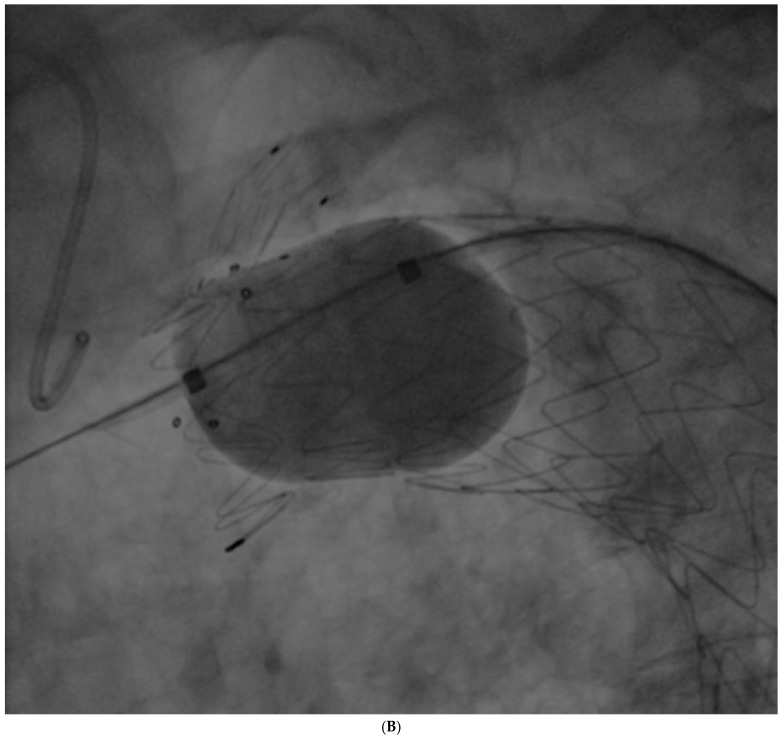

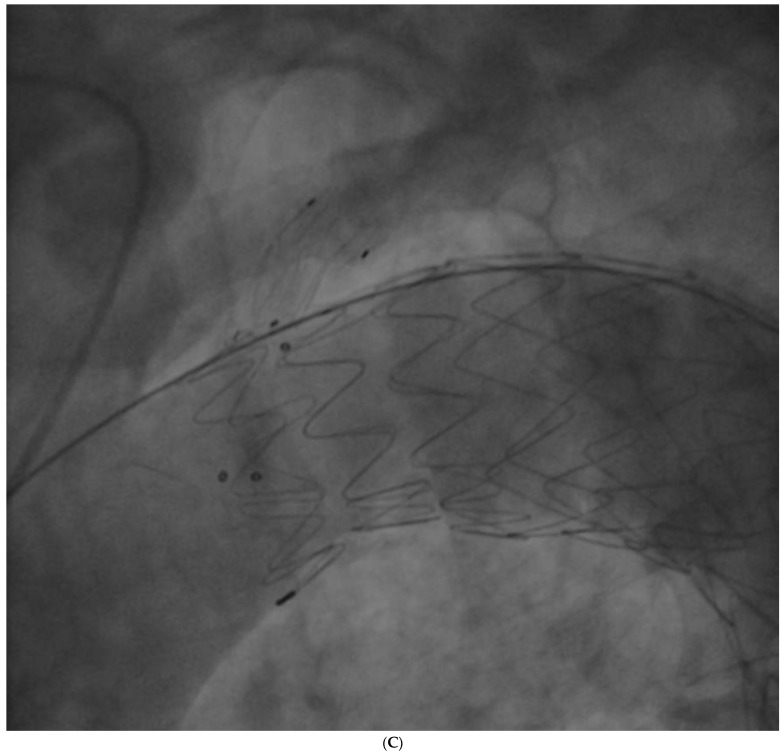
(**A**)—Type I endoleak observed after graft deployment; black arrows show endoleak. (**B**)—Balloon inflation for type I endoleak treatment. (**C**)—Final result after balloon inflation; no endoleak is observed.

The procedure was performed in deep sedation. A 6 F pigtail was advanced through a right radial arterial access into the ascending aorta for aortic angiographies; then a 10 F right femoral arterial access was obtained with the pre-implantation of two percutaneous closure systems, and subsequently. upgraded to 18 F. Afterwards, an 8 F left brachial arterial access was obtained; a guidewire was advanced through the brachial access into the femoral access, forming a brachial–femoral loop; a dedicated catheter was advanced on the guidewire; then, an extra stiff guidewire (0.035″) was advanced through femoral access to ascending aorta, and the branch guidewire was positioned in the catheter introduced from the brachial access. A Castor 38 mm × 32 mm × 200 mm stent graft with a 12 mm × 25 mm branch for LSA was positioned and released at PAU level. The final angiography showed the exclusion of PAU, but also a small type 1 endoleak (Figure 5A) treated successfully with some balloon dilatations in the proximal graft lumen (Reliant Balloon, Figure 5B,C). This finding is likely due to graft malapposition caused by an enlarged ascending aorta and aortic arch. There were no complications during the procedure. The patient was discharged with anti-hypertensive treatment (candesartan 16 mg, hydrochlorothiazide 12.5 mg, amlodipine 5 mg), aspirin, plus clopidogrel (for 3 months, followed by low-dose aspirin only).

After 1 year, the patient continued to be asymptomatic, and the CT angiography showed a stable PAU exclusion and absence of any type of endoleak.

## 5. Discussion

The prevalence of acute aortic diseases is increasing in Western countries. The patients are generally older, male, hypertensive and smokers. Up to now, little is known about PAU, and there are no randomized trials evaluating treatment options. Strategies for the open repair of PAU are varied, depending on the location and presentation of the PAU. There are many factors that make endovascular repair more attractive than open surgery:

- PAU often occurs in older patients with atherosclerotic disease, and these patients are at high risk during open surgery [9];
- Open repair more often leads to death, stroke, myocardial infarction, bowel infarction or aorto-oesophageal fistulation [10];
- Aortic tissue in patients with PAU may be thin and damaged, making open graft repairs difficult [11].

PAU has classically been described in the descending thoracic aorta; however, a significant number may also be found in the aortic arch [8]. The management of PAU differs depending on its location. European guidelines suggest that surgery should be considered for Type A PAU [12], and American guidelines state that emergency surgery should be considered in lesions affecting the ascending aorta [11].

When PAU is located in the aortic arch or just distally to LSA, patients may require open intervention. Since patients (as cases 2 and 3) are often asymptomatic, the risks of an invasive treatment could overcome clinical benefits. To manage this problem, we used a single-branched aortic stent graft (Endovastec™ Castor™). The Castor single-branch stent graft is designed to exclude entry tear and preserve the LSA by using a branch section. It is made by a main body and a side that originates 5 to 30 mm from the prosthesis proximal edge. The branched stent graft is constructed of woven polyester fabric sewn to self-expanding Nitinol stents, without a proximal or distal bare stent. The delivery system consists of an outer sheath coated with a low friction, hydrophilic layer, and an inner soft polyester fabric sheath encapsulating the aortic graft trunk and the branch section, which are individually folded. The aortic graft trunk is folded with loops of thread and a nickel–titanium wire [13]. This endovascular prosthesis can present different lengths and diameters and it can be customized according to patient’s aortic anatomy; thus, it is crucial to obtain angio-CT images, not only to plan vascular access and procedural steps, but also to implant a prosthesis as similar as possible to a native aortic arch and descending aorta anatomy.

In our case series, the procedures were performed under deep sedation (1 patient) or general anesthesia (2 patients). In some cases, regional or local anesthesia can be used. The choice is based on several factors, such as preoperative comorbidities, hemodynamic stability, procedure time, expected blood loss, and the need for transesophageal echocardiography (TEE), which influence this decision. General anesthesia is currently the most popular anesthetic technique for TEVAR because it provides a secure airway with controlled ventilation, facilitates TEE monitoring, and ensures immobility [14].

We believe that the most important intraprocedural aspect could be the positioning of the landing zone on the proximal edge of the prosthesis; in fact, the operator needs to check the correct alignment of radiopaque markers at the LSA level from different radiological points of view before releasing the device in order to properly expand the main body and the side branch. The antero-posterior and left anterior oblique radiological projections are very useful for graft positioning and deployment. Once the graft is positioned, little manipulation of endoprosthesis is generally needed to reduce neurological complication and intima injury.

Endoleaks represent possible complications in positioning endovascular prosthesis. In our case series, one patient developed a type I endoleak, likely because the presence of dilated ascending aorta; this intraprocedural finding was treated with some balloon inflation. The real endoleaks occurrence with Castor endoprosthesis is unknown, mainly due to a lack of large registries or clinical trials. Recently, a Chinese perspective trial [13] (including 73 patients with B-type aortic dissections) showed good results using this type of endoprosthesis; in this study, the mortality rate was 6% and periprocedural complications were very low during a median follow-up of 5 years; notably, there were four intraoperative endoleaks, and there was no evidence of endoleaks during follow-up. These data appear encouraging, especially when compared to other techniques for preserving supra-aortic vessels, such as the “chimney technique”. In fact, the incidence of endoleaks reported in the “chimney technique” was about 18%, either treated with coiling, additional stenting or spontaneously resolved [15]. The possible explanation of this finding could be that, in the “chimney technique”, an ideal radial force should be exerted from the aortic endograft so as not to compromise the chimney graft, while maintaining adequate wall apposition; however, when this force is too weak, this situation lead to endoleaks development.

At present, there is no general consensus about the precise use of antithrombotic agents in the endovascular treatment of AAS. Data from the literature published over the past decade showed a trend of administering and prolonging dual antiplatelet therapy (DAPT); DAPT in particular is preferred to single-antiplatelet therapy (SAPT) in subjects who have undergone endovascular aortic aneurysm exclusion with the snorkel technique, treated with increased numbers of visceral vessels [16]. We suggest continuing with a dual antiplatelet therapy (generally with a low dose of acetylsalicylic acid and clopidogrel) for 3 months after the implantation, followed by SAPT long-life. This antithrombotic regimen was effective in our cases since no evidence of endoprosthesis lumen thrombosis was observed. In fact, the first 90 days after graft deployment are considered the most vulnerable period for thrombosis occurrence. When a graft stenosis is observed during early post-deployment angiographies, stenting areas of under-expansion represents the correct strategy to minimize acute and subacute graft thrombosis [17]. With regard to safety, this short-term DAPT did not cause any haemorrhagic complications, even in case 1 for whom risk of bleeding was high (old age and previous intracranial hematoma).

Since the first implantation in Poland in October 2020, this kind of device has rarely been used in Europe. In our limited experience, the short-term clinical and angiographic results are encouraging, and we consider the procedure feasible and safe, but a longer follow-up and larger randomized controlled trials are needed. The possibility of having the device off the shelf will also guarantee the same simplicity and safety in acute treatments.

## 6. Conclusions

With ageing and the increase in atherosclerotic burden in patients, acute aortic syndromes are increasing. Nowadays, PAU treatment strategies are not well-established and conservative management is not always the best option. In high-surgical-risk patients with particular anatomical PAU location, a full percutaneous procedure using a single-branched stent graft avoids sternotomy and extracorporeal circulation and could reduce mortality or complications, especially in asymptomatic patients with isolated PAU. According to our experience, this full percutaneous approach should be widely used in the future, but these observational data need to be confirmed in large randomized controlled trials.
